# Microbial Disease Spectrum Linked to a Novel IL-12Rβ1 N-Terminal Signal Peptide Stop-Gain Homozygous Mutation with Paradoxical Receptor Cell-Surface Expression

**DOI:** 10.3389/fmicb.2017.00616

**Published:** 2017-04-13

**Authors:** Thais Louvain de Souza, Regina C. de Souza Campos Fernandes, Juliana Azevedo da Silva, Vladimir Gomes Alves Júnior, Adelia Gomes Coelho, Afonso C. Souza Faria, Nabia M. Moreira Salomão Simão, João T. Souto Filho, Caroline Deswarte, Stéphanie Boisson-Dupuis, Dara Torgerson, Jean-Laurent Casanova, Jacinta Bustamante, Enrique Medina-Acosta

**Affiliations:** ^1^Núcleo de Diagnóstico e Investigação Molecular, Laboratório de Biotecnologia, Universidade Estadual do Norte FluminenseCampos dos Goytacazes, Brazil; ^2^Faculdade de Medicina de CamposCampos dos Goytacazes, Brazil; ^3^Laboratório de Biologia do Reconhecer, Universidade Estadual do Norte FluminenseCampos dos Goytacazes, Brazil; ^4^Hospital Municipal Ferreira MachadoCampos dos Goytacazes, Brazil; ^5^Pronto Atendimento UNIMEDCampos dos Goytacazes, Brazil; ^6^Hospital Escola Álvaro AlvimCampos dos Goytacazes, Brazil; ^7^Laboratory of Human Genetics of Infectious Diseases, Institut National de la Santé et de la Recherche MédicaleParis, France; ^8^Laboratory of Human Genetics of Infectious Diseases: Mendelian Predisposition, Imagine Institute, Paris Descartes UniversityParis, France; ^9^St. Giles Laboratory of Human Genetics of Infectious Diseases, The Rockefeller UniversityNew York, NY, USA; ^10^Department of Medicine, University of California San FranciscoSan Francisco, CA, USA; ^11^Pediatric Hematology-Immunology Unit, Necker Hospital for Sick Children, Assistance Publique Hôpitaux de ParisParis, France; ^12^Howard Hughes Medical Institute, The Rockefeller UniversityNew York, NY, USA; ^13^Study Center of Primary Immunodeficiencies, Assistance Publique Hôpitaux de Paris, Necker Hospital for Sick ChildrenParis, France

**Keywords:** IL-12Rβ1 deficiency, Mendelian susceptibility to mycobacterial diseases, impaired receptor, IFN-γ, signal peptide, founder stop-gain mutation

## Abstract

Patients with Mendelian Susceptibility to Mycobacterial Diseases (MSMD) exhibit variable vulnerability to infections by mycobacteria and other intramacrophagic bacteria (e.g., *Salmonella* and *Klebsiella*) and fungi (e.g., *Histoplasma, Candida, Paracoccidioides, Coccidioides*, and *Cryptococcus*). The hallmark of MSMD is the inherited impaired production of interferon gamma (IFN-γ) or the lack of response to it. Mutations in the interleukin (IL)-12 receptor subunit beta 1 (*IL12RB1*) gene accounts for 38% of cases of MSMD. Most *IL12RB1* pathogenic allele mutations, including ten known stop-gain variants, cause IL-12Rβ1 complete deficiency (immunodeficiency-30, IMD30) by knocking out receptor cell-surface expression. *IL12RB1* loss-of-function genotypes impair both IL-12 and IL-23 responses. Here, we assess the health effects of a rare, novel *IL12RB1* stop-gain homozygous genotype with paradoxical IL-12Rβ1 cell-surface expression. We appraise four MSMD children from three unrelated Brazilian kindreds by clinical consultation, medical records, and genetic and immunologic studies. The clinical spectrum narrowed down to Bacillus Calmette-Guerin (BCG) vaccine-related suppurative adenitis in all patients with one death, and recrudescence in two, histoplasmosis, and recurrence in one patient, extraintestinal salmonellosis in one child, and cutaneous vasculitis in another. In three patients, we established the homozygous Trp7Ter predicted loss-of-function inherited genotype and inferred it from the heterozygote parents of the fourth case. The Trp7Ter mutation maps to the predicted IL-12Rβ1 N-terminal signal peptide sequence. BCG- or phytohemagglutinin-blasts from the three patients have reduced cell-surface expression of IL-12Rβ1 with impaired production of IFN-γ and IL-17A. Screening of 227 unrelated healthy subjects from the same geographic region revealed one heterozygous genotype (allele frequency 0.0022) vs. one in over 841,883 public genome/exomes. We also show that the carriers bear European ancestry-informative alleles and share the extended CACCAGTCCGG *IL12RB1* haplotype that occurs worldwide with a frequency of 8.4%. We conclude that the novel *IL12RB1* N-terminal signal peptide stop-gain loss-of-function homozygous genotype confers IL-12Rβ1 deficiency with varying severity and early-onset age through diminished cell-surface expression of an impaired IL-12Rβ1 polypeptide. We firmly recommend attending to warning signs of IMD30 in children who are HIV-1 negative with a history of adverse effects to the BCG vaccine and presenting with recurrent *Histoplasma* spp. and extraintestinal *Salmonella* spp. infections.

## Introduction

Here, we report four pediatric cases that exemplify the microbial and clinical spectra of infectious diseases associated with the impaired production of the Th_1_ signature cytokine interferon-gamma (IFN-γ) in subjects with Mendelian Susceptibility to Mycobacterial Diseases (MSMD; Zerbe and Holland, 2005; Kutukculer et al., 2006; de Beaucoudrey et al., 2010; Zahid et al., 2014). These comprised attenuated bacillus Calmette-Guerin (BCG) vaccine-related suppurative adenitis in all patients with one death, and recrudescence in two, histoplasmosis, and recurrence in one patient, extraintestinal salmonellosis in one child, and cutaneous vasculitis in another. Our subjects share a history of adverse events to the BCG vaccine in the first year of life. BCG vaccine adverse effects are a major warning sign of primary immunodeficiency disorder of the IFN-γ axis as the underlying cause (de Beaucoudrey et al., 2010; Carneiro-Sampaio et al., 2011; Costa-Carvalho et al., 2014; Abolhassani et al., 2015). The four children are from three apparently unrelated kindreds, living in 60-km^2^ proximity near the city of Campos dos Goytacazes, situated in the northern region of the State of Rio de Janeiro, Brazil. Moreover, the parents of Case 1 are first-degree relatives. Therefore, we based the framework of investigation on the hypothesis of a rare homozygous loss-of-function alteration in the interleukin (IL)-12 receptor subunit beta 1 (*IL12RB1*) gene occurring in a small population.

## Kindred A

### Case 1

White male, born in 1999, the only child of first-degree consanguineous parents. BCG-vaccinated during the first month of life. At age 3 months, he developed suppurative axillary adenitis, ipsilateral to the BCG vaccine site, and was treated with Isoniazid (10 mg/kg/day), with regression of the lymphadenopathy. At age 9 months, he was hospitalized due to fever, sweating, failure to thrive, severe anemia and hepatic and splenic enlargements. Findings included normal chest X-ray; cultures of blood and urine negative for bacteria; hemoglobin 6 g/dL; leukocytes 2,700 cells/mm^3^ (0% eosinophils, 1% bands, 2% monocytes, 47% neutrophils, and 50% lymphocytes); 40,000 platelets/mm^3^; normal AST (36 IU/L) and ALT (20 IU/L). A myelogram demonstrated erythroid and platelet hypoplasias and a blockage in granulocyte development. He was treated with Isoniazid (10 mg/kg/day) and with Ceftriaxone (100 mg/kg /day) and received two blood transfusions. Mother and child were both HIV-1 negative. After 7 days, he was transferred to the intensive treatment unit, with respiratory failure and died after massive pulmonary bleeding.

## Kindred B

### Case 2

**Figure 1:**
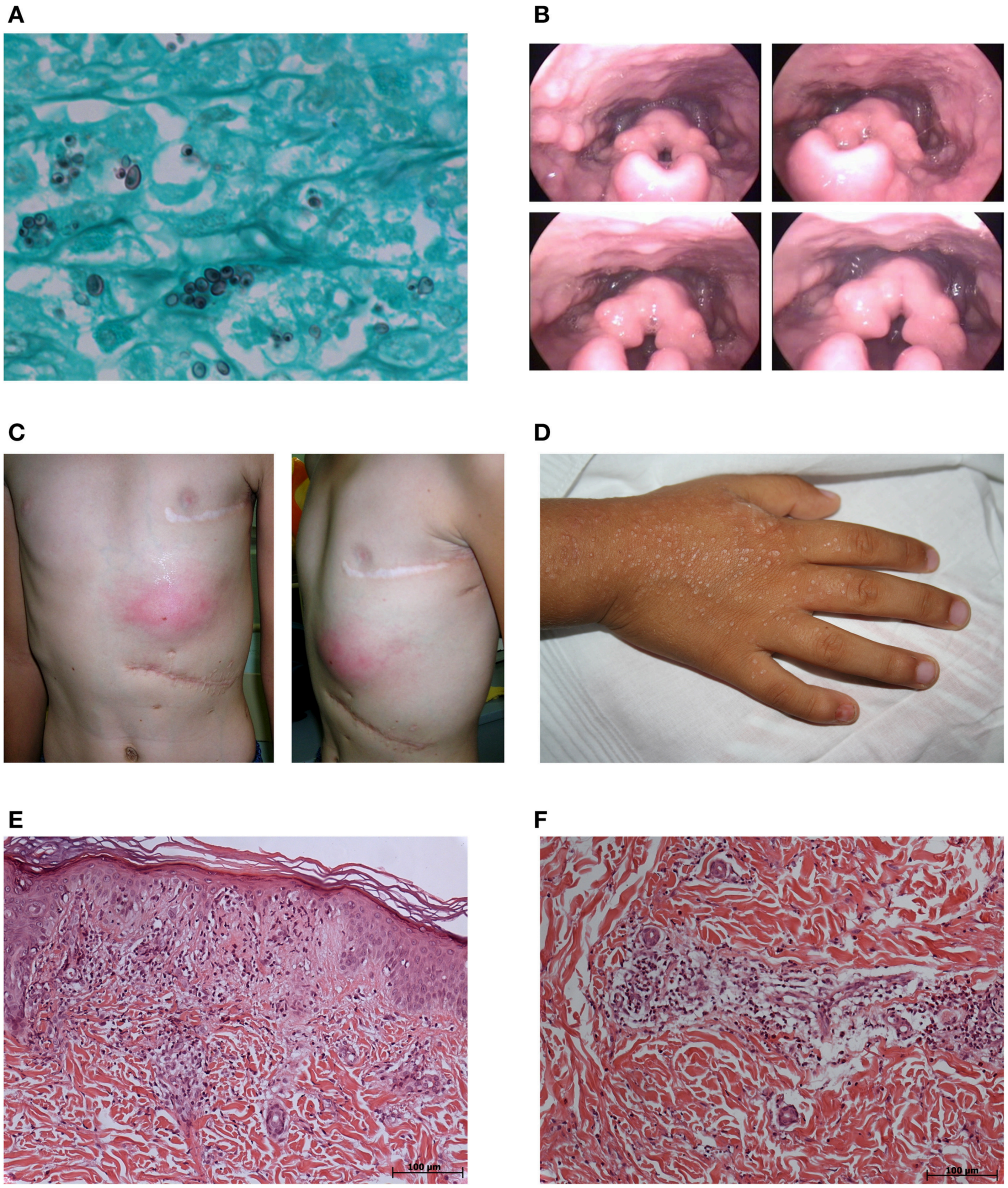
**Clinicopathological findings in the affected children**. Case 2: **(A)** Presence of many intracellular, small, narrow base, budding, and darkly pigmented yeast-like cells (1–5 mm diameter) in the lymph node (Grocott stain, original magnification × 1,000). **(B)** Screenshots of video laryngoscopy showing bilateral granulomatous lesions of aryepiglottic folds. Case 3: **(C)** Frontal and lateral photographs of the left infracostal large abscess. Case 4: **(D)** Photograph of the pruritic, maculopapular erythematous rash in the back of the right hand. **(E)** Hyperkeratosis and regional atrophy associated with basal vacuolization and focal necrosis in addition to perivascular and interstitial inflammatory mixed infiltrate with mild nuclear fragmentation in the erythematous maculopapular rash on the left hand (hematoxylin-eosin stain, × 100). **(F)** Perivascular and interstitial inflammatory mixed infiltrates with neutrophils in karyorrhexis, lymphocyte, and eosinophils in the erythematous lesions of the left limb (hematoxylin-eosin stain, × 100).

White male, born in 1993, the first child of a non-consanguineous couple. BCG-vaccinated during the first month of life. At age 1 year, he presented with suppurative axillary adenopathy ipsilateral to the BCG vaccination site. He was treated with Isoniazid (10 mg/kg/day) for 6 months, with good clinical evolution. At age 13 years, he had cervical lymphadenopathy and fever. Histoplasmosis was confirmed by histopathology (Figure 1A) and was treated with ketoconazole (400 mg/day) with excellent response. At age 18 years, he had a relapse of histoplasmosis manifested by bulky cervical lymphadenopathy, hoarseness, dysphagia, and fever. Video laryngoscopy revealed bilateral granulomatous lesions of the aryepiglottic folds (Figure 1B). Immunological findings included hyper-IgE (17,300 and 17,937 IU/mL in two separate tests; Reference Value, RV: <87); IgG = 2,790 mg/dL (RV: 549–1584), IgA = 225 mg/dL (RV: 61–348), IgM = 106 mg/dL (RV: 23–259); CD4 = 350 cells/mm^3^ (41.5%), CD8 = 269 cells/mm^3^ (17.2%), CD4/CD8 = 1.30. Histoplasmosis recurrence was treated with Itraconazole (600 mg/day) for 2 years. He is currently healthy without prophylaxis.

### Case 3

White male, born in 2001, a full sibling of Case 2. BCG-vaccinated during the first month of life. At age 4 months, he had regional suppurative adenitis ipsilateral to the BCG vaccine site and was treated with Isoniazid (10 mg/kg/day) for 6 months with good clinical response. At age 5 years, when his older brother (Case 2) was diagnosed with histoplasmosis, he presented with multiple cervical lymphadenopathies and fever. He was treated with ketoconazole (400 mg/day). Treatment was interrupted 7 months later without recovery. At age 6 years, he was hospitalized with cervical lymphadenopathies and was treated with Amphotericin B without regression. At age 8 years, he was hospitalized with asthenia, anorexia, abdominal pain and cervical adenopathies, and evolved hepatic and splenic enlargements, respiratory distress, persistent fever, pericardial and pleural collections and splenic abscess, which demanded drainage and resection, respectively. Laboratory studies showed leukocytosis (26,900 cells/mm^3^; 14% bands, 80% segments, 5% lymphocytes) with normal platelet count (297,000/mm^3^), AST (38 U/L), ALT (25 IU/L), AP (342 IU/L), and altered albumin (2.30 g/dL), globulin (3.60 g/dL); total bilirubin (7.80 mg/dL); and direct bilirubin (6.60 mg/dL). The Wade, PAS and Giemsa stains of lymph nodes and spleen were negative. The PAS and Wade stains of the pericardial membrane were negative. The culture of bone marrow aspirate was positive for *Salmonella choleraesuis*. Besides antibacterial therapy (40 mg/kg/day of Vancomycin and 30 mg/kg/day of Meropenem), Rifampin (10 mg/kg/day), Isoniazid (10 mg/kg/day), and Pyrazinamide (20 mg/kg/day) were administered due to polyserositis and the unconfirmed possibility of household contacts with tuberculosis. Immunological evaluation revealed: hyper-IgE (902 and 2,848 IU/mL (RV at 87 IU/ml) in two separate evaluations as well as elevated values for IgG = 3,150 mg/dL (RV: 572–1,474), IgA = 943 mg/dL (RV: 34–305), IgM = 223 mg/dL (RV: 32–208), and T cell markers (CD4 = 955 cells/mm^3^ (17%), CD8 = 1,348 cells/mm^3^ (24%), CD4/CD8 = 0.7. Three months after suspension of the tuberculostatics, there was a recrudescence of cervical lymphadenopathy with fistulization despite the absence of fever and inflammatory signs. Cultures of ganglion aspirate were negative for bacteria, acid-fast bacilli, and fungi. Histopathology of cervical lymph nodes was negative for intracellular microbes. He received experimental treatment with Trimethoprim/Sulfamethoxazole for 5 months with complete recovery and prophylaxis with the same medication for 6 months. After the interruption of prophylaxis, he developed a left infracostal large abscess (Figure 1C), which was drained and found negative on culture. He was empirically prescribed with Ciprofloxacin (40 mg/kg/day) for *Salmonella* and Clarithromycin (15 mg/kg/day) for mycobacteria for 6 months. He is currently on prophylaxis with Trimethoprim/Sulfamethoxazole and is free of symptoms.

## Kindred C

### Case 4

White male, born in 2010, the only child of a non-consanguineous couple. BCG-vaccinated during the first month of life. At age 6 months, he developed axillary adenopathy ipsilateral to the BCG vaccine site and was treated with Isoniazid (10 mg/kg/day) for 6 months. Four months later, adenopathy recurred with fistulization and was successfully managed with Isoniazid (10 mg/kg/day) and Rifampin (10 mg/kg/day) for 6 months. Cultures from an adenopathy puncture sample were negative for bacteria, mycobacteria, and fungi. At age 5 years, presented with ankle arthritis, no fever, and non-pruritic erythematous lesions in the lower limbs and buttocks, which did not disappear with compression, and progressive flares for shorter periods during 4 months, which included one full body episode, with a clinical diagnosis of leukocytoclastic vasculitis. He also evolved a pruritic, maculopapular erythematous rash in the neck and the back of both hands (Figure 1D), with a clinical diagnosis of pityriasis. Laboratory studies showed leukocytosis (14,980 cells/mm^3^), ESR 25 mm/h (RV 0–20 mm/h), and LDH 730 IU/L (RV 200–480 IU/L). Immunological findings included elevated IgE (934 IU/ml [RV at 52 IU/ml]), IgG = 5,650 mg/dL (RV: 504–1,465), IgA = 340 mg/dL (RV: 27–195), and IgM = 537 mg/dL (RV: 24–210); CD4 = 1,280 cell/mm^3^ (23.26%); CD8 = 2,080 cells/mm^3^ (37.79%); and CD4/CD8 = 0.62. Normal renal function, with no proteinuria or hematuria. Positive for rheumatoid factor (512 IU/mL; RV <8 IU/mL). Negative for antinuclear antibodies. Histopathology of an erythematous maculopapular rash on the left hand revealed hyperkeratosis and regional atrophy associated with basal vacuolization and focal necrosis in addition to perivascular and interstitial inflammatory mixed infiltrates with mild nuclear fragmentation (Figure 1E) and, thus, inconsistent with the diagnosis of pityriasis. Histopathology of the erythematous lesions of the left limb showed inflammatory mixed perivascular, and interstitial infiltrates with neutrophils in karyorrhexis, lymphocytes, and eosinophils (Figure 1F), confirming the diagnosis of leukocytoclastic vasculitis. Positive Widal H agglutination test (flagella B reactive). Blood and stool cultures negative for bacteria. He was treated with Ciprofloxacin (40 mg/kg/day) for 2 weeks with regression of lesions and no flares. He is currently on Trimethoprim/Sulfamethoxazole prophylaxis.

**Figure 2:**
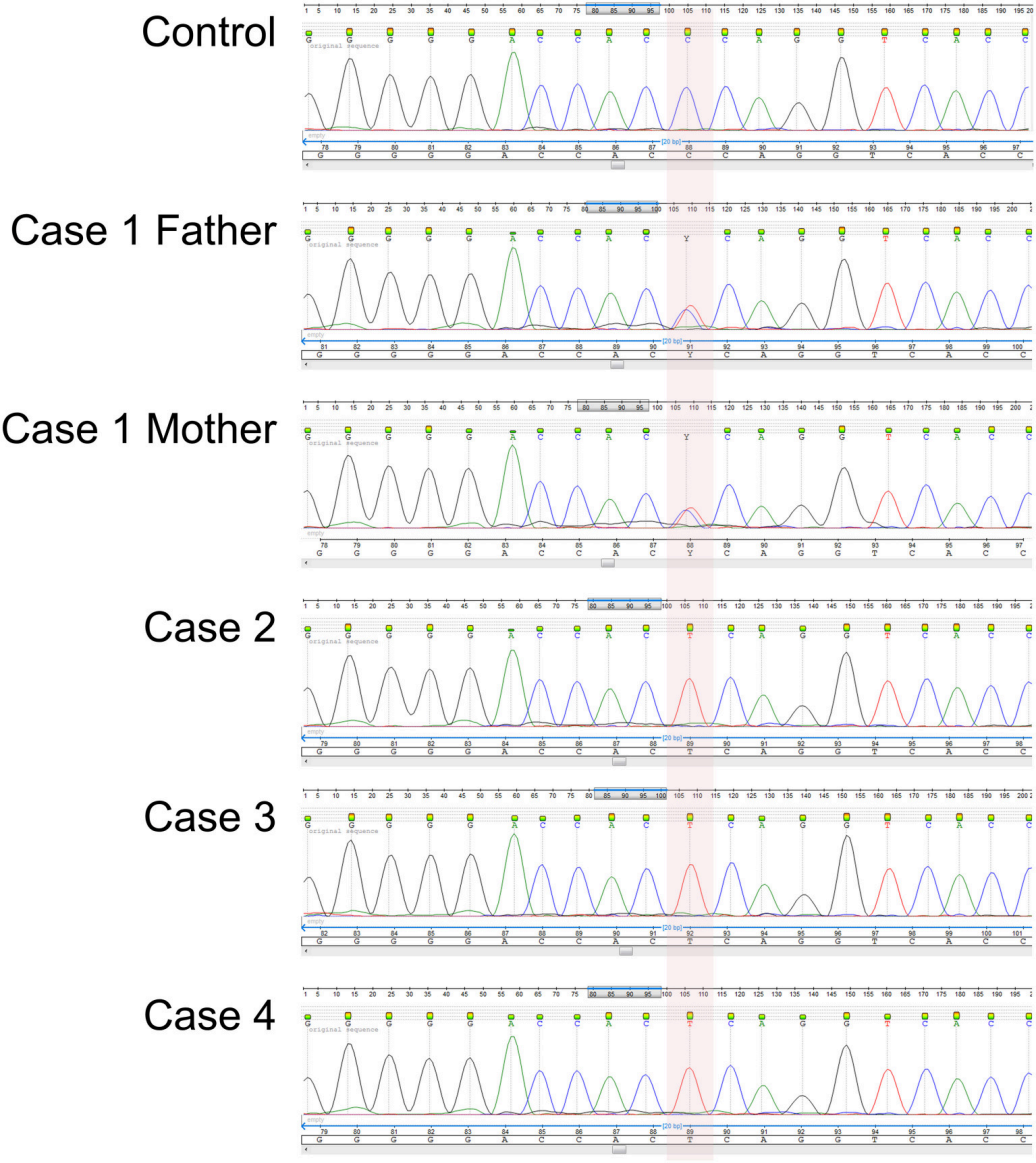
**Electropherogram of the novel ***IL12RB1*** predicted loss-of-function genotype confirmed by Sanger sequencing**. The area shaded in pink indicates the homozygous Trp7Ter predicted loss-of-function genotype for the SNP variant rs150172855 (c.21G>A/p.Trp7Ter/TGG ⇒ TGA) identified in Cases 2, 3, and 4. As exemplified here with Case 1, the parents of all four cases were heterozygous carriers. The stop-gain mutation is located in exon 1 within the predicted coding region of the signal peptide. Shown are the sequencing traces for the forward strand of DNA.

We sequenced the exons of the *IL12RB1* gene in Cases 2, 3, and 4 (no DNA sample was available from Case 1) and found all three to be homozygous for the autosomal recessive Trp7Ter predicted loss-of-function single nucleotide polymorphism (SNP) c.21G>A/p.Trp7Ter/TGG ⇒ TGA (Figure 2). We next sequenced the parents from the three unrelated kindreds and confirmed them all to be heterozygous for the stop-gain allele variant (Figure 2). Henceforth, we assume that Case 1 was homozygous for the stop-gain allele variant. We also genotyped a population subset of 227 unrelated individuals from the northern region of the State of Rio de Janeiro and established that the Trp7Ter predicted loss-of-function allele variant occurs at a frequency of 1 in 454 chromosomes (allele frequency of 0.0022). We note that the unrelated carrier in this subset lives in a rural area of the district of Travessão, 20.1 km from the city of Campos dos Goytacazes. Thus, we identified three unrelated couples being at high risk of recurrence in future pregnancies.

The Trp7Ter stop-gain mutation is absent in 841,883 genotyped or sequenced genomes and whole exomes worldwide in public databases (NHLBI GO Exome Sequencing Project, 2012; Sulem et al., 2015; Chen et al., 2016; Lek et al., 2016; Narasimhan et al., 2016; Supplementary Data). However, a heterozygous carrier of the stop-gain mutation was identified in the Severe Asthma Research Program (SARP) at a frequency of 0.0005 (1 out of 2,056 chromosomes; Moore et al., 2007; Torgerson et al., 2012) and deposited into dbSNP under rs150172855. The subject is of self-reported European ancestry. To assess the genetic ancestry of this individual, we ran a Principal Component Analysis using genome-wide SNP genotypes with other subjects from the same cohort study (Torgerson et al., 2012). These included African American and European American population subsets as well as the HapMap phase 3 populations (International HapMap et al., 2010). Consistent with the participant's self-reported European ancestry, this individual clustered with the HapMap population with Northern and Western European ancestry (CEU) and other persons of self-reported European ancestry from the SARP (Supplementary Figure S1). We also determined that Cases 2, 3, and 4 share the CACCAGTCCGG (forward strand) extended homozygous genotype, which spans 27.3 kb across the *IL12RB1* gene (Supplementary Figure S2, Supplementary Table S1, and Supplementary data). Moreover, the three probands are homozygous for seven out of eight tested informative SNP variant alleles of European ancestry (Supplementary Table S2, and Supplementary data).

**Figure 3:**
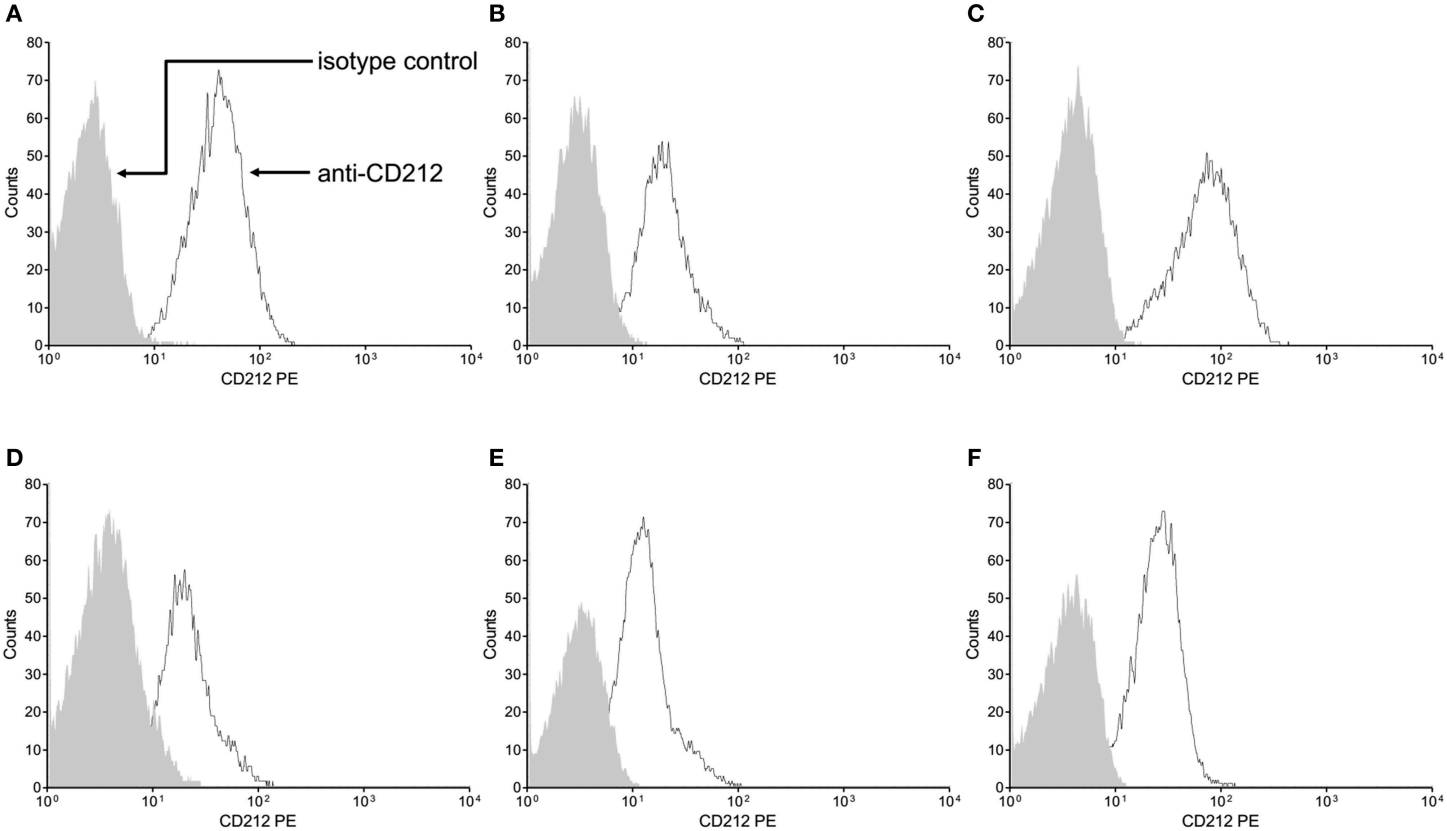
**The Trp7Ter stop-gain mutation at the signal peptide sequence does not knock out cell-surface expression of the IL-12Rβ1 polypeptide**. Cell-surface expression of IL-12Rβ1 protein antigen in peripheral blood mononuclear cells was ascertained by flow cytometry in a representative non-carrier control subject **(A)**, and the heterozygous carrier mothers of case 2 **(B)** and case 4 **(C)** and the homozygous Trp7Ter predicted loss-of-function cases 2 **(D)**, 3 **(E)**, and 4 **(F)**. The PHA-T-cell blasts were stained with phycoerythrin (PE)-labeled mouse 2.4E6 monoclonal antibody anti-Human CD212 (IL-12Rβ1), or PE-labeled matched control isotype. The arrows point to the matched control isotype and CD212 specific staining intensities depicted as the shaded and open areas under the curves, respectively. In all subjects, stimulated cells exhibited increased fluorescence intensity, reflecting the presence of the CD212 specific IL-12Rβ1 antigen at the cell surface, independently of the occurrence of the stop-gain mutation. For comparison, the expression mean percentage of positive cells in seven non-carrier controls was 88.2.

**Figure 4:**
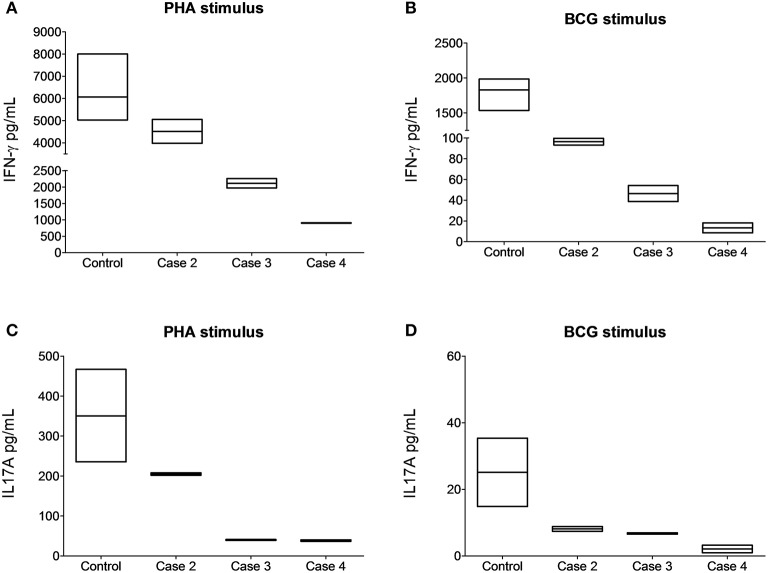
**The cell-surface-expressed IL-12Rβ1 protein in the affected children is reduced and functionally impaired**. Cytokine-specific Cytometric Bead Array analyses revealed reduced production of IFN-γ in response to PHA **(A)** and BCG lysate **(B)** and of IL-17A in response to PHA **(C)** and BCG lysate **(D)** stimuli in cultured peripheral blood monocular cells from the subjects homozygous for the *IL12RB1* Trp7Ter predicted loss-of-function mutation vs. the control subjects. For comparison, in five control non-carriers, the IFN-γ mean expression values were 6,063 and 1,830 pg/mL upon stimulation with PHA or BCG lysate, respectively. For IL17-A cytokine, the mean expression values were 350.3 and 25.10 pg/mL under the same stimuli. Quantification assays were done on samples taken at least 4 months after remission (3 years for case 2, 4 months for case 3, and 2 years for case 4). Boxes, interquartile ranges; horizontal lines within boxes, median values.

We showed *ex vivo* that the stop-gain mutation at the signal peptide coding sequence, paradoxically, does not knock out cell-surface expression of the IL-12Rβ1 polypeptide (Figure 3). As observed in a non-carrier control subject (Figure 3A), and the two heterozygous carrier mothers (kindred B, Figure 3B and kindred C, Figure 3C), the IL-12Rβ1 protein antigen is present in the cell-surface of PHA-blasts from Case 2 (Figure 3D), Case 3 (Figure 3E), and Case 4 (Figure 3F). Importantly, the *ex vivo* and *in vitro* levels of the cell-surface expressed IL-12Rβ1 polypeptide are diminished in these children, and the receptor activity is affected because the production of IFN-γ in response to phytohemagglutinin (PHA; Figure 4A) and BCG lysate (Figure 4B) and of IL-17A in response to PHA (Figure 4C) and BCG (Figure 4D) stimuli is impaired vs. the control subjects. Worth of note, PHA-blasts from Case 2 produced >4,000 pg/mL of IFN-γ, which is close to the levels observed in the controls (Figure 4A), and contrasting to the situation in the younger affected brother (Case 3). There are reports of close to normal and even higher levels of IFN-γ production in PHA- or BCG-blasts of a few other patients with IMD30 (de Jong et al., 1998; Caragol et al., 2003; Lichtenauer-Kaligis et al., 2003; Moraes-Vasconcelos et al., 2005; Schepers et al., 2013; Ramirez-Alejo et al., 2014).

IL-12Rβ1 is a type I transmembrane receptor chain; thus, it has an extracellular N-terminal domain topology. Interestingly, the Trp7Ter mutation maps to within the predicted 23-amino acid N-terminal signal peptide coding sequence. By imputation, the Trp7Ter stop-gain mutation creates either an exonic acceptor splice silencer (ESS) site, an acceptor splice site or a donor splice site [Supplementary Figure S3 with potentially deleterious effect(s) through RNA mis-splicing]. Whether the Trp7Ter stop-gain mutation is deleterious through exon 1 partial in-frame skipping in the *IL12RB1* gene or there is transcriptional initiation from potentially alternative non-AUG start sites downstream of the annotated AUG (i.e., Val9, Leu16, or Leu17; Supplementary Figure S3) is still unclear.

## Background

MSMD comprises 19 complete or partial primary immunodeficiencies of the IFN-γ-mediated immunity (IFN-γ axis). Mutations in seven autosomal genes (*IFNGR1, IFNGR2, IL12B, IL12RB1, IRF8, ISG15, STAT1*) and two X-linked genes (*IKBKG* and *CYBB*) caused MSMD (Bustamante et al., 2014). The hallmark of MSMD is the impaired production of IFN-γ or a lack of response to it. MSMD patients exhibit variable vulnerability to early and often life-threatening infections with intramacrophagic bacteria (*Mycobacterium* spp., *Salmonella* spp., and *Klebsiella* spp.) and fungi (*Candida* spp., *Paracoccidioides* spp., *Coccidioides* spp., *Cryptococcus* spp., and *Histoplasma* spp.; Moraes-Vasconcelos et al., 2005; de Beaucoudrey et al., 2010). Adverse events are an important warning sign of MSMD in the context of vaccination with BCG, BCG-itis (local or regional disease), or BCG-osis (disseminated disease; de Beaucoudrey et al., 2010; Costa-Carvalho et al., 2014; Abolhassani et al., 2015).

A recent database update compiled 77 pathogenic allele variants in the *IL12RB1* gene on chromosome 19p13 in 211 patients (de Beaucoudrey et al., 2010; van de Vosse et al., 2013), which accounts for 38.2% of cases of MSMD. *IL12RB1* loss-of-function impairs both IL-12 and IL-23 responses. Most pathogenic allele mutations in the *IL12RB1* gene cause complete IL-12Rβ1 deficiency (immunodeficiency-30, IMD30) without cell-surface expression of the receptor. However, in homozygous carriers of the c.230T>C, c.632G>C, c.700+362_1619-944del, and p.(Pro233_Glu234insVGLVLIA) variants (Scheuerman et al., 2007; van de Vosse et al., 2013; Ramirez-Alejo et al., 2014), the cell-surface expression of the IL-12Rβ1 polypeptide is reduced (Lichtenauer-Kaligis et al., 2003; Fieschi et al., 2004). There are only ten reported stop-gain mutations linked to IMD30 with information on cell-surface expression (de Jong et al., 1998; de Beaucoudrey et al., 2010; Boisson-Dupuis et al., 2011; van de Vosse et al., 2013). Significantly, all ten stop-gain variants knockout cell-surface expression of the receptor.

## Discussion

We describe the health effects of the *IL12RB1* inactivation by the novel N-terminal signal peptide Trp7Ter stop-gain, predicted loss-of-function inherited homozygous genotype in three children from two apparently unrelated Brazilian kindreds and inferred the same genotype in a fourth case from a third geographically proximate kindred. All four probands have a history of BCG adverse events (suppurative adenitis in all four and recurrence in two patients) at an early age, three of them exhibited diminished cell-surface expression of the IL-12Rβ1 polypeptide and had impaired production of IFN-γ and IL-17A in response to *in vitro* stimuli. The genotype-phenotype correlations observed in these four children are a hallmark of MSMD (de Beaucoudrey et al., 2010). Besides the complications with the BCG vaccine, clinical features varied in breadth with different age of onset, and were associated mainly with *Histoplasma* spp. and *Salmonella* spp. infections. In all four cases, the health effects necessitated hospitalization with surgical interventions in two subjects. We believe that the cause of death of Case 1 was BCG-osis because the severe manifestations appeared shortly after the interruption of the intermittent use of Isoniazid and a lack of response to Ceftriaxone, which excluded a *Salmonella* infection.

Except for candidiasis, fungal diseases in IMD30 are rare. Reports in endemic countries are one case each by *Histoplasma* spp. in India (de Beaucoudrey et al., 2010), *Paracoccidioides brasiliensis* in Brazil (Moraes-Vasconcelos et al., 2005), *Cryptococcus neoformans* in Thailand (Jirapongsananuruk et al., 2012), and two cases by *Coccidioides* spp. in Palestine (Vinh et al., 2011). None of our four subjects had candidiasis. Case 2 had histoplasmosis and relapsed 5 years after the first episode. In the first episode, he was treated with ketoconazole. With *Histoplasma* spp. recrudescence, he was successfully managed with Itraconazole, following the updated guidelines (Wheat et al., 2007). Worth noting, in immunocompetent patients, histoplasmosis has been successfully managed using ketoconazole (Faiolla et al., 2013). Cases of disseminated histoplasmosis have been reported in individuals with IFN-γR1 deficiency (IMD27B; Zerbe and Holland, 2005), which indicates that the IFN-γ response is necessary for the control of histoplasmosis (Clemons et al., 2000). In Brazil, the number of reported cases of histoplasmosis is small, with male gender, HIV-1 infection, and rural environment being the main risks factors mentioned (Faiolla et al., 2013). All four our patients live in a rural environment, but only Case 2 had histoplasmosis.

*Salmonella* infections in patients with MSMD are mainly caused by non-typhoid serotypes and involve severe extraintestinal manifestations that include lymphadenopathy, bacteremia, and septicemia (MacLennan et al., 2004). Cases 3 and 4 presented with different clinical manifestations of *Salmonella* infections. Case 3 had sepsis by *S. choleraesuis* and was successfully managed with appropriate antibiotics and splenectomy. Recurrence of suppurative cervical adenopathies and the infracostal abscess in this patient were also probably related to *Salmonella* infection because the early onset of BCG disease has a protective role against reactivation of latent mycobacteria or a new infection (de Beaucoudrey et al., 2010) and the adequate management with Ciprofloxacin in the last episode.

Case 4 had recurrent cutaneous vasculitis. Leukocytoclastic vasculitis by *Salmonella* is a rare condition in MSMD patients. There are only three cases reported in IMD30 (Kutukculer et al., 2006; Sanal et al., 2006; Filiz et al., 2014). The last episode of cutaneous vasculitis in Case 4 was a full-body flare, which ruled out Henoch-Schönlein purpura. Because the child was positive for antibodies to *Salmonella* flagellar antigens and there was complete remission after treatment with Ciprofloxacin, we believe *Salmonella* infection caused the vasculitis in this subject. Other infectious diseases, use of medicines, connective tissue disorders, and malignancies were excluded.

Elevated IgE occurs in atopic dermatitis and allergic diseases (Woodfolk et al., 2015). Cases 2 and 3 evolved IgE >2,000 IU/mL but presented no symptoms of atopy, such as asthma or eczema, or mild allergy. Hyper-IgE syndrome (Job syndrome) was excluded in these children because of the clinical course. Elevated IgE occurred in some reported cases of IMD30, none of which were due to loss-of-function mutations (Altare et al., 1998; Caragol et al., 2003; Carvalho et al., 2003; Moraes-Vasconcelos et al., 2005; Luangwedchakarn et al., 2009), and in one case of Chronic Granulomatous Disease, which is a type of MSMD characterized by mycobacterial granuloma formation (Patiroglu et al., 2013). In the later case, IgA deficiency was also reported and no mutations in the *STAT3* and *DOCK8* genes, characteristic in hiper-IgE syndrome, were found. We believe the elevated IgE in Cases 2 and 3 reflects a state of chronic antigenic stimulation by the intracellular microbes. Thus, for children with no signs of atopy, we propose that elevated IgE is an important warning sign to investigate the narrow spectrum of MSMD. Measurement of IgE levels should be valuable also for patients seen in countries where BCG vaccination is not universal. Overall, the causative *IL12RB1* mutations imply that Th_1_ signature cytokines have no major regulatory effect on Th_2_ cells in IMD30 patients (Döffinger et al., 1999). However, the Th_1_/Th_2_ polarization paradigm needs to be directly assessed in the IMD30 cases reported this far, by measuring IL-4, IL-5, IL-10, and IL-13 levels, and assessing the influence of the deficiency in the expression levels of the transcription factors T-bet, GATA-3 and RORγt.

We found that Cases 2, 3, and 4 were homozygous for the extended CACCAGTCCGG *IL12RB1* haplotype. In the 1,000 Genomes Project database (Sudmant et al., 2015), this haplotype occurs at a rate of 8.4%, with 47.5, 18.2, and 11.6% of the haplotype counts, being distributed among individuals of European, American and African ancestry, respectively (Supplementary Table S3). Thus, the pathogenic Trp7Ter germline allele likely represents a founder mutation among individuals from the northern region of the State of Rio the Janeiro, Brazil, who carried an extended haplotype that is more common in European populations. These findings are significant for both estimating disease risk in the population and genetic counseling for the affected families.

## Concluding remarks

The novel Trp7Ter predicted loss-of-function homozygous *IL12RB1* genotype was linked to the paradoxical cell-surface expression of an impaired IL-12Rβ1 polypeptide with health effects that varied in severity and early-onset age mainly regarding susceptibility to *Mycobacteria* spp., *Histoplasma* spp., and *Salmonella* spp. Our study expands the narrow spectrum of clinical manifestations observed in pediatric IMD30 by including histoplasmosis recurrence. We urge on health care professionals to evaluate IMD30 in HIV-1 negative children with a history of adverse effects to the BCG vaccine and presenting with recurrent *Histoplasma* spp. and extraintestinal *Salmonella* spp.

## Ethics statement

This study was carried out in accordance with the recommendations of the Brazilian National Ethics Committee CONEP with written informed consent from all subjects. All subjects gave written informed consent in accordance with the Declaration of Helsinki. The protocol was approved by the CONEP (national approval registry CAAE no. 35385714.0.0000.5244). The subjects were included from a pilot, observational, exploratory, qualitative study entitled *Investigation of the molecular and genetic basis of the severe adverse events to the BCG vaccine in the context of the Chronic Granulomatous Disease and the Mendelian Susceptibility to Mycobacteria Disease*. A major goal of the study was to determine the rates of adverse events to the BCG vaccine in children as warning signs of primary immunodeficiencies in otherwise healthy children. The study involved physical and history examinations of subjects, laboratory testing for primary immunodeficiencies, management of episodes of infectious diseases, and genetic testing and counseling. Sampling was by convenience or accessibility, in subjects who presented with or have a history of adverse events to the BCG vaccine. Peripheral blood samples from participating controls and the three Brazilian families were collected with written informed consent. For infants and children, a surrogate consent procedure was used, whereby the next of kin or a legally authorized representative approved in writing on behalf of the participants.

## Author contributions

TL, RF, JC, JB, and EM: conceived and designed experiments. TL: performed genotyping, sequencing, analyzed the data. TL and JA: performed flow cytometry studies. TL, VG, and EM: performed genotyping of extended haplotypes. RF, AG, AS, NM, and JS: recruited, attended patients. CD, SB, JC, and JB: performed genetic screening and sequencing. DT: performed principal component analysis. EM: carried out comprehensive computational analysis, contributed biological samples, reagents, materials, made figures, and wrote the manuscript.

## Funding

This work was supported by grants from the Fundação de Amparo à Pesquisa do Estado do Rio de Janeiro (BR) (http://www.faperj.br/) [grant numbers E-26/110.775/2011, E-26/111.715/2012 and E26/010.001036/2015 to EM and E26/010.000892/2015 to RF], and from Conselho Nacional de Desenvolvimento Científico e Tecnológico (BR) (http://cnpq.br/) [grant number 301034/2012-5 to EM]. TL received a graduate fellowship (grant number 1121200877) from Universidade Estadual do Norte Fluminense Darcy Ribeiro (BR) (http://www.uenf.br/). VG received an undergraduate fellowship (grant number E-26/202.426/2015) from Fundação de Amparo à Pesquisa do Estado do Rio de Janeiro (BR). The agencies had no role in the study design, data collection, and analysis, decision to publish, or preparation of the manuscript.

### Conflict of interest statement

The authors declare that the research was conducted in the absence of any commercial or financial relationships that could be construed as a potential conflict of interest.
