# Supplementary material for: Microbial Disease Spectrum Linked to a Novel IL-12Rβ1 N-Terminal Signal Peptide Stop-Gain Homozygous Mutation with Paradoxical Receptor Cell-Surface Expression
Source: Front Microbiol. 2017 Apr 13;8:616. doi: 10.3389/fmicb.2017.00616 (PMC5389975; doi:10.3389/fmicb.2017.00616)
Supplement: Supplementary file 4 [file DataSheet1.doc]

Supplementary Material

**Microbial Disease Spectrum Linked to a Novel IL-12Rβ1 N-Terminal Signal Peptide Stop-Gain Homozygous Mutation with Paradoxical Receptor Cell-Surface Expression**

**Thais Louvain de Souza**, **Regina Célia de Souza Campos Fernandes**, **Juliana Azevedo da Silva**, **Vladimir Gomes Alves Júnior**, **Adelia Gomes Coelho**, **Afonso Celso Souza Faria**, **Nabia Maria Moreira Salomão Simão**, **João Tadeu Damian Souto Filho**, **Caroline Deswarte**, **Stéphanie Boisson-Dupuis**, **Dara Torgerson**, **Jean-Laurent Casanova**, **Jacinta Bustamante**, **Enrique Medina-Acosta^*^**

*** Correspondence**: Enrique Medina-Acosta: quique@uenf.br

## SUPPLEMENTARY DATA: MATERIAL AND METHODS

**DNA extraction and Sanger sequencing**

DNA genomic of control samples and index case families were extracted using the commercial Illustra Blood Genomic Prep Mini Spin Kit (GE Healthcare, Little Chalfont, UK) and stored at -20ºC. We used exon-based PCR products to survey for rare mutants located in the *IL12RB1* gene by Sanger sequencing methodology from genomic DNA from the available subjects.

**Isolation and culture of mononuclear cells**

Fresh peripheral blood mononuclear cells (PBMC) were isolated using Ficoll-Paque PLUS density gradient media (GE Healthcare Bio-Sciences Corp., NJ, USA). Cultivation of 1x10^6^ cells/well was in RPMI medium supplemented with 2mM L-glutamine, 100 units of penicillin, 200μg/mL streptomycin and 10% fetal bovine serum. Cells were stimulated *ex vivo* for 72h with 1mg/mL phytohemagglutinin (PHA) (Sigma-Aldrich, MI, USA) or 0.2mg/mL BCG lysate (Moreau strain, Fundação Ataulpho de Paiva, RJ, Brazil).

**Detection of IL-12Rβ1**, **IFN-γR1**, **and quantification of cytokines**

Culture supernatants were stocked at -80^o^C for quantification of the human IFN-γ and IL17A cytokines using cytometric bead arrays (CBA # 558269 and # 562143, respectively, from Becton, Dickinson and Company, BD Biosciences, San Jose, CA, USA). Membrane expression of IL-12Rβ1 (phycoerythrin (PE)-PE-labelled mouse 2.4E6 monoclonal anti-Human CD212 antibody and PE-labelled mouse IgG1 isotypic control) and IFN-γR1 (PE-labelled mouse anti-Human CD119 and PE-labelled mouse IgG2b isotypic controls) were carried by flow cytometry. Antibodies were purchased from Biolegend, CA, USA. For whole blood: 50μL blood cells were lysed according to the protocol reported in (Vowells et al., 1995), followed by the addition of 5-20μL of each antibody in separate tubes (volumes adjusted according to the manufacturer recommendations). Samples were incubated for 30 minutes at 4°C in the dark and washed twice with PBS containing 2% fetal bovine and 0.1% NaN3, by centrifugation at 500xg for 3 minutes at 4°C. Cells were suspended in 450μL of the same buffer. For culture cells, each well (1x10^6^ cells/100μL) was washed with 200μL wash buffer as above and incubated with each antibody, diluted according to the manufacturer instructions, for 30 minutes in the dark at 4°C. After incubation, cells were washed twice with wash buffer and suspended in 450μL. Preparations were analyzed using an FACSCalibur Flow Cytometry platform (BD Biosciences).

**Genotyping and allele frequency determination**

For the population subset of 227 unrelated healthy individuals from the northern region of the State of Rio de Janeiro, DNA typing for the rs150172855 (G>A) SNP was using a DdeI restriction enzyme fragment length polymorphism-based quantitative ﬂuorescence PCR assay specific for the rs150172855-T allele. The amplification primers are in Supplementary Table S. The 5´-forward primer was modified with the fluorochrome FAM for fragment detection. PCR reactions were prepared in 12.5µL containing 4 to 8 ng of genomic DNA, 1.25µL of 10x PCR buffer (200 mM Tris-HCl, pH 8.4, 500 mM KCl), 1.25 µL 200 mM dNTP mix, 0.75 µL of 50 mM MgCl_2_, 0.8 µM of each primer and 0.05 µL of Taq Gold DNA polymerase (5U/µL; Invitrogen Corporation, San Diego, CA, USA). Thermal cycling conditions were as follows: 95⁰C for 11 min (1 cycle); 94⁰C for 1 min; 59⁰C for 1 min; 72⁰C for 1 min (28 cycles) and 60⁰C for 60 min (1 cycle) on a Gene AmpPCRsystem 9700 (Applied Biosystems, Foster City, CA, USA). The 229 bp amplimer was then digested with 1U DdeI (New England Biolabs, Ipswich, Massachusetts, United States) in 5.5 µL final volume at 37⁰C for four hr. Allele proﬁles were determined on an ABI 310 Prism Genetic Analyzer (Applied Biosystems). Data were analyzed using GeneScan Analysis 3.7 and Genotyper 3.7 software (Applied Biosystems). The amplimer carrying the rs150172855*A nonsense mutation is susceptible to digestion by the restriction enzyme *Dde*I and thus is represented by a final 113 bp product, whereas the ancestral rs150172855*G allele is refractory to digestion and a 229 bp product represents it. In this assay, a heterozygous carrier has the 113 bp/229 bp genotype. We validated the heterozygous carriers by single nucleotide primer extension (SNuPE) specific assay and Sanger sequencing using juxtaposed elongation primer (Supplementary Table S1). The amplimers were analyzed by high-resolution capillary electrophoresis (Alves da Silva et al., 2016).

**Genotyping of *IL12RB1* extended haplotypes**

We used a SNuPE multiplex assay to genotype the *IL12RB1*extended haplotype comprising the SNPs rs3833286 (indel), rs3746190, rs11575935, rs401502, rs375947, rs845381, rs11575934, rs11575926, rs147215816, rs17887176, rs436857 (Supplementary Table S1) in the 3 kindreds and in 20 unrelated individuals from the same geographic region. The extended haplotype spans 27.3 kb. We used the 2,504 phase genotypes from the 1000Genomes project (Sudmant et al., 2015), available at the Ensembl browser (Cunningham et al., 2015) (http://www.ensembl.org/; [accessed 04/01/2016]), to determine the frequency of the 11 SNP extended haplotype found in our subjects. To determine the physical length and the genetic context of the extended haplotype, we map the SNPs using a custom track at the UCSC Genome Browser hg19 graphical interface (Kent et al., 2002) (http://genome.ucsc.edu).

**Genotyping with ancestry-informative markers**

We retrieved information for ancestry-informative markers (AIMs) from genomic data sets, available at the public AncestrySNPminer browser (Amirisetty et al., 2012) (https://research.cchmc.org/mershalab/AncestrySNPminer/home.php). We selected SNPs (Supplementary Table S2) with the highest allele frequency asymmetry observed between African and European subsets as reported by dbSNP (Sherry et al., 2001). We used SNuPE-specific assays to genotype the carriers of the Trp7Ter stop-gain mutation with the AIMs. Amplimers were analyzed by high-resolution capillary electrophoresis (Alves da Silva et al., 2016).

**Prediction of changes in splicing due to the stop-gain mutation**

We analyzed the reference and the mutated sequences for the N-terminal signal peptide of the *IL12RB1* gene for predicted changes in splicing using the online software tools Human Splicing Finder (Desmet et al., 2009) (http://www.umd.be/HSF3/; [accessed 04/01/2016]) and Mutation Taster (Schwarz et al., 2014) (http://www.mutationtaster.org/; [accessed 04/01/2016]).

**Population stratification by principal components analysis**

To model ancestry differences based on allele frequency differences between cases and controls, we performed principal components analysis using the standalone SmartPCA program in EIGENSTRAT (Price et al., 2006). Participants in the Severe Asthma Research Program (SARP) included those with mild to severe asthma and controls (Moore et al., 2007; Torgerson et al., 2012b). Genotyping of participants in the SARP was performed on the Illumina 1Mv1 as previously described (Li et al., 2012; Torgerson et al., 2012a). Genotypes were merged with those from the HapMap phase 3 study (International HapMap et al., 2010) and pruned for linkage disequilibrium using PLINK (Purcell et al., 2007). Acces to genotypes was through data sharing, approved by Dr. Deborah A. Meyers (Wake Forest School of Medicine, North Carolina, C, USA) on behalf of the SARP.

**Public databases of genetic variants**

We surveyed public databases of genetic variants, and found that the stop-gain mutation is absent in 138,632 unrelated individuals from the Genome Aggregation Database (gnomAD, release version 2.0 r) (Lek et al., 2016), 104,220 Icelanders (Sulem et al., 2015), 6,503 individuals from the NHLBI GO Exome Sequencing Project (NHLBI GO Exome Sequencing Project, 2012), 3,222 British Pakistani-heritage adults with high parental relatedness (Narasimhan et al., 2016), and even 589,306 genotyped or sequenced genomes and whole exomes worldwide (Chen et al., 2016).

**SUPPLEMENTARY FIGURES**

**FIGURE S1**. **Principal component analysis of African American** (**AfAm**) **and European American** (**EurAm**) **participants in the SARP and the HapMap phase 3 populations**. Shown are the first two principal components generated using SmartPCA in EIGENSTRAT (Price et al., 2006). A single heterozygous carrier of the rs150172855*A stop-gain mutation in the SARP study (Moore et al., 2007; Torgerson et al., 2012a) (indicated by a circle cross) clustered with individuals of European ancestry. A description of the population codes for the HapMap phase 3 is in Supplementary Table S3.

**FIGURE S2**. **The physical chromosomal positions of the SNPs used to identify the *IL12RB1* extended haplotype**. From top to bottom: Ideogram of chromosome 19, the exon-intron organization of the two major *IL12RB1* reference isoforms (dark blue boxes), the physical location of the rs3833286 (indel), rs3746190, rs11575935, rs401502, rs375947, rs845381, rs11575934, rs11575926, rs147215816, rs17887176, rs436857 SNPs used for genotyping, and the extended CACCAGTCCGG haplotype identified in the three Brazilian kindreds. The stop-gain rs150172855*A allele is highlighted in red within the extended C**A**ACCAGTCCGG haplotype. Screenshot generated using custom tracks in the UCSC Genome Browser hg19 (http://genome.ucsc.edu).

**FIGURE S3**. **Predicted deleterious effects of the Trp7Ter mutation**. Paradoxical cell-surface expression of an impaired IL-12Rβ1 polypeptide from the mutated *IL12RB1* RNA may involve exon 1 partial in-frame skipping (mis-splicing) or transcriptional initiation from potentially alternative in-frame non-AUG start sites downstream of the annotated AUG. In the annotated IL-12Rβ1 there is a predicted N-terminal signal peptide sequence with a putative cleavage site after amino acid 23 (reference DNA), being 21 residues encoded by exon 1. The novel Trp7Ter mutation maps to within the signal peptide sequence converting the tryptophan (W) reference codon TGG at position 7 into the stop codon TGA (mutant DNA). By imputation, the mutation creates an exonic acceptor splice silencer (ESS) (ACCTGAGTGGTCCCCCTCCTCTTCCTCTTC), an acceptor splice site (tggtgacctgagTG) and a donor splice site (TGAgtggtc). Alternative non-AUG sites encoding are indicated, with CTG (Leu16 and Leu17) being the most efficient in mammals (Mehdi et al., 1990).

**SUPPLEMENTARY TABLES**

**Table S1**. Information about the SNPs used in the determination of the extended *IL12RB1* haplotypes.

**Table S2**. Information about the Ancestry Informative Markers used in this study and genotypes of the probands, homozygous for the Trp7Ter stop-gain mutation.

**Table S3**. Phased extended haplotypes across the *IL12RB1* gene found in 2,504 individuals from the 1,000 Genome Project and population distribution of the CACCAGTCCGG extended haplotype genotyped in the affected Brazilian children.

**References**
