# Supplementary figures and images for: Microbial Disease Spectrum Linked to a Novel IL-12Rβ1 N-Terminal Signal Peptide Stop-Gain Homozygous Mutation with Paradoxical Receptor Cell-Surface Expression
Source: Front Microbiol. 2017 Apr 13;8:616. doi: 10.3389/fmicb.2017.00616 (PMC5389975; doi:10.3389/fmicb.2017.00616)

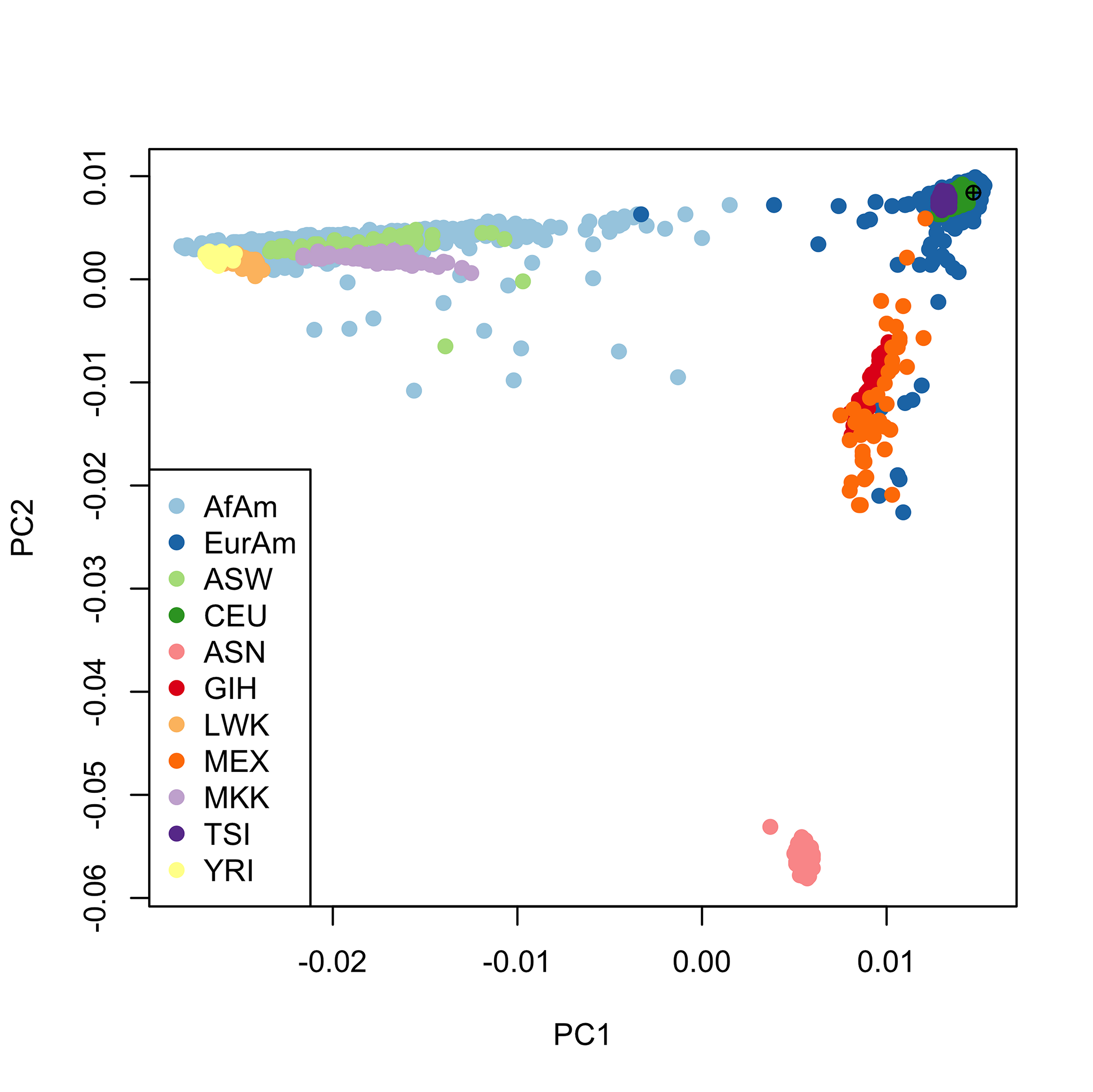

Supplement: Supplementary file 5 [file Image1.TIFF]

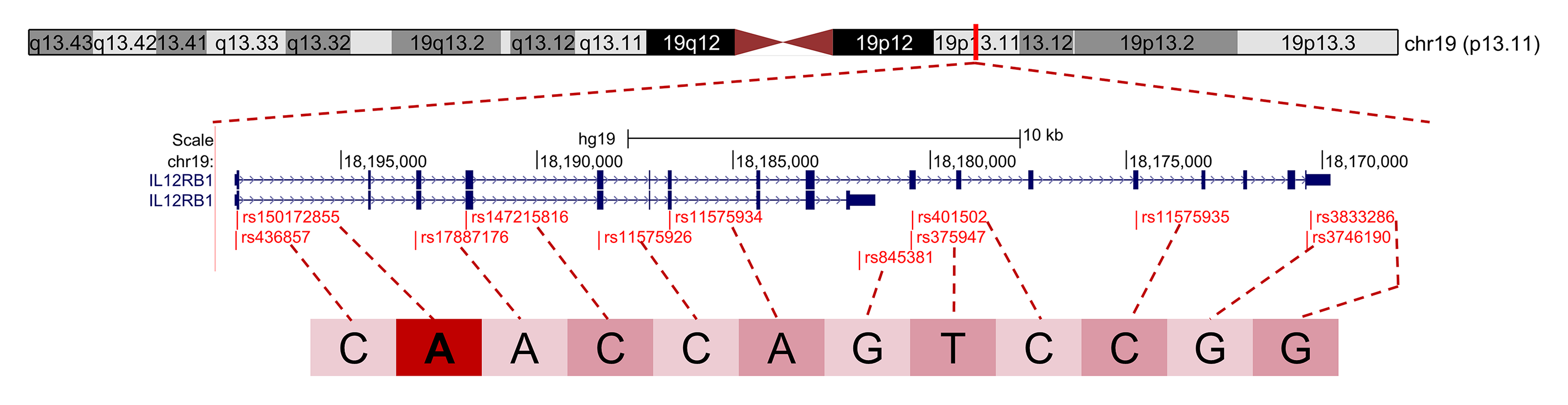

Supplement: Supplementary file 6 [file Image2.TIF]

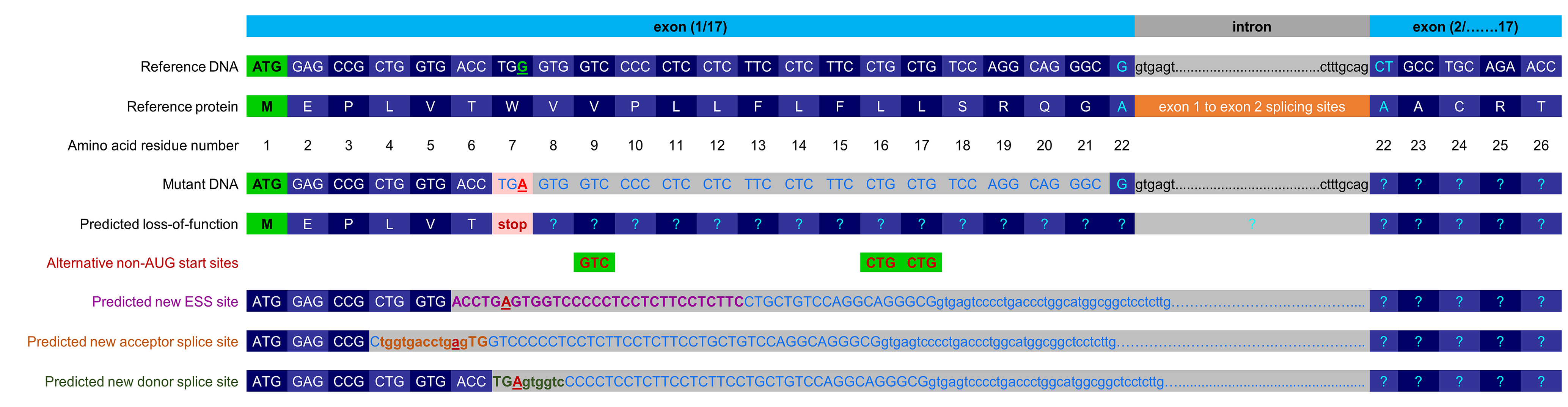

Supplement: Supplementary file 7 [file Image3.TIF]
